# A Minireview on BET Inhibitors: Beyond Bromodomain Targeting

**DOI:** 10.3390/biomedicines13030594

**Published:** 2025-03-01

**Authors:** Mikhail S. Iudin, Yuri M. Khodarovich, Anna M. Varizhuk, Vladimir B. Tsvetkov, Vyacheslav V. Severov

**Affiliations:** 1 Lopukhin Federal Research and Clinical Center of Physical-Chemical Medicine of Federal Medical Biological Agency, 119435 Moscow, Russia; yudin.ms75@gmail.com (M.S.I.); annavarizhuk@gmail.com (A.M.V.); v.b.tsvetkov@gmail.com (V.B.T.); 2 Moscow Center for Advanced Studies, 123592 Moscow, Russia; 3 Shemyakin-Ovchinnikov Institute of Bioorganic Chemistry, Russian Academy of Sciences, 117997 Moscow, Russia; khodarovich@mail.ru; 4 Research and Educational Resource Center for Cellular Technologies of The Peoples’ Friendship University of Russia, 117198 Moscow, Russia; 5 Center for Mathematical Modeling in Drug Development, Sechenov First Moscow State Medical University, 119991 Moscow, Russia

**Keywords:** BET proteins, BET inhibitors, BET phosphorylation sites, extra-terminal domain, BRD4, IDR

## Abstract

Bromodomain and extra-terminal domain (BET) proteins are epigenetic readers that recognize the histone acetylation code and play a critical role in regulating gene transcription. Dysregulation of BET proteins is associated with a number of pathologies, including cancer, inflammation-related metabolic disorders, etc. BET proteins can also be hijacked by some viruses and mediate latent viral infections, making BET proteins promising targets for therapeutic intervention. Research in this area has mainly focused on bromodomain inhibition, with less attention paid to other domains. Bromodomain inhibitors have great potential as anticancer and anti-inflammatory drug candidates. However, their broad-spectrum impact on transcription and potential cross-reactivity with non-BET bromodomain-containing proteins raise concerns about unforeseen side effects. Non-bromodomain BET inhibitors hold promise for gaining better control over the expression of host and viral genes by targeting different stages of BET-dependent transcriptional regulation. In this review, we discuss recent advances in the development of non-bromodomain BET inhibitors, as well as their potential applications, advantages, and perspectives.

## 1. Introduction

Bromodomains (BDs) are acetyl-lysine binding domains commonly found in chromatin-associated proteins. To date, 61 bromodomain-containing proteins have been identified in the human proteome. They are grouped into 46 families and eight structural groups [1]. Among these, the bromodomain and extra-terminal (ET) domain family (BET) is the most widely studied due to its therapeutic relevance. BET proteins serve mainly as readers of the histone epigenetic code, activators of transcription, and regulators of 3D chromatin structure [2]. Their dysregulation is involved in many pathologies, such as inflammation, cancer, metabolic disorders, neurodegenerative diseases, etc. [3,4,5,6,7].

The BET family consists of four proteins: BRD2, BRD3, BRD4, and testis/ovary-specific BRDt [8]. Each of them contains two BDs (BD1 and BD2) and an ET domain, whose major functions include interactions with histones and transcription regulators, respectively [9]. The highly conserved tandem BDs and the ET domain are interspersed with intrinsically disordered regions (IDRs). The remarkably long C-terminal IDR of the full-length BRD4 isoform (BRD4L, the main isoform), complemented by a C-terminal motif (CTM) that folds into an α-helix, is associated with a high propensity for phase transitions [10]. BRDt also contains a relatively long C-terminal IDR with CTM, but its phase transition propensity has not yet been verified. The main part of the C-terminal IDR is absent in the short BRD4 isoforms BRD4S(a) and BRD4S(b) generated by alternative splicing. The latter slightly longer isoform has a distinct C-terminus. It has only been described in U2OS cells and is therefore of limited therapeutic relevance, so BRD4S(a) will be referred to simply as BRD4S in the following sections.

The structures, functions, and interactors of BET proteins in normal and pathological conditions, as well as BET-related signaling pathways, have been reviewed elsewhere [1,5,8,9,11,12,13]. Particular attention has been paid to the BD-dependent common function of BET proteins as epigenetic readers. Accordingly, BET BDs are among the most prominent therapeutic targets.

Since 2010, when the first two BET bromodomain inhibitors (BBDi), namely, **JQ1** [14] and **I-BET** [15], were discovered, there has been spectacular growth in the development of new small-molecule BBDi. Over 30 BBDi have entered clinical trials for the treatment of inflammation, cardiovascular diseases and cancer [16,17]. However, some of them have shown mixed outcomes, and most of the ongoing trials (<20, according to https://clinicaltrials.gov/, accessed on 20 February 2025) are still in Phase I/II. Recent advances in this field are summarized in several elegant reviews [2,16,18]. Known BBDi can be classified according to their chemical structure, selectivity, and mechanism of action. The vast majority of BBDi act as histone competitors and block the acetyl-lysine binding pocket of BD. Notable BBDi variants with a more advanced mechanism of action are BET-PROTACs (PROteolysis-TArgeting Chimeras). They recruit an E3 ligase, which marks BET proteins for degradation.

Regarding selectivity, the following classification can be outlined:

- Pan-BET inhibitors that do not discriminate between BET family members or the two BDs of a given BET;
- Tyrosine kinase and BET dual inhibitors that exploit cross-family BD homology and show promise as anticancer drug candidates due to the synergistic effect;
- BD1- or BD2-selective inhibitors [18];
- Bivalent BBDi that target both BDs of a given BET and typically show improved selectivity compared to monovalent analogues;
- Selective inhibitors that distinguish between BET family members (e.g., BRD4-specific inhibitors, which are particularly promising anticancer candidates).

As concerns the chemical structure, most BBDi are small molecules with minimal contact surface area, which explains their limited selectivity. Peptide-based inhibitors can outperform small-molecule compounds, since their relatively large size, structural complexity and diversity [19] allow for tight and specific binding to BDs. Notable examples are the cyclic peptides developed by Patel et al. [20]. These exceptionally specific BBDi were selected from a library of over 10^12^ compounds using mRNA display-based random nonstandard peptides integrated discovery. However, the strategies for intracellular delivery of these cyclopeptides have not been fully developed so far, and their intracellular distribution awaits clarification.

**Figure 1:**
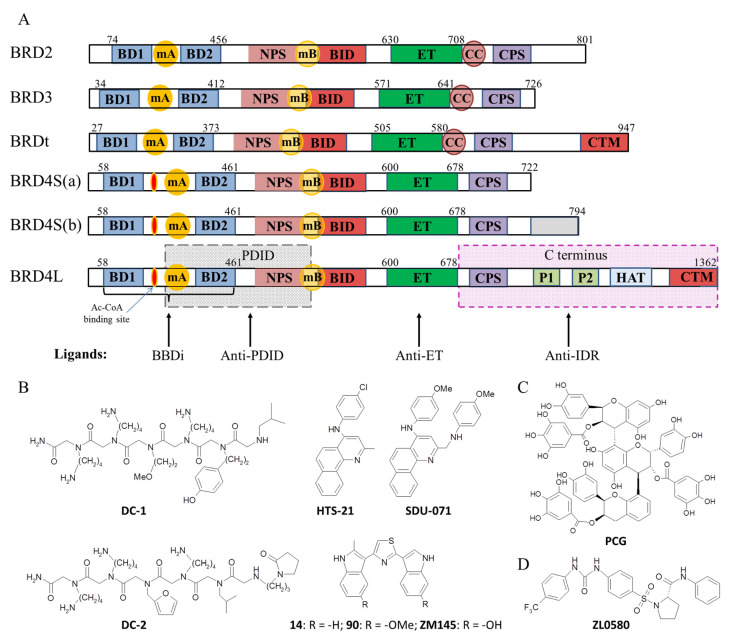
BET proteins and noncanonical BET inhibitors. (**A**) Domain organization and isoforms of BET proteins. BET: bromodomain and extra-terminal; mA and mB: motifs A and B; NPS: N-terminal cluster of phosphorylation sites; PDID: phosphorylation-dependent interaction domain; BID: basic residue-enriched interaction domain; ET: extra-terminal domain; CC: coiled-coil motif; CPS: C-terminal cluster of phosphorylation sites; P1 and P2: Pro-rich sites; HAT: histone acetyl transferase catalytic motif; BRD4L: long isoform of BRD4; CTM: C-terminal motif; BRD4S(a/b): short isoforms a/b of BRD4. (**B**) Structures of anti-PDID ligands targeting phosphorylated NPS. (**C**) Structure of the anti-IDR ligand. (**D**) Structure of the noncanonical BBDi.

Although BBDi show anticancer efficacy in preclinical models, especially in combination with some other classes of antitumor drugs due to synergistic effects [1,2,3], most of them have pronounced toxicity at high doses [21,22,23,24,25]. The toxicity of BBDi-PROTACs is typically due to on-target but off-tissue effects [21,22], and can be minimized by advanced delivery approaches [23]. In the case of pan-BBDi, the toxicity likely results from off-target effects within the BET family, disruption of the extensive network of BET interactions, and inhibition of non-BET BD-containing proteins [24,25]. The limitations of BBDi can be partially overcome by targeting other BET domains or motifs, such as, ET, IDRs, the basic residue enriched interaction domain (BID), or others (Figure 1A). These domains may be equally important for the recruitment of chromatin remodelers and transcription factors to acetylated chromatin regions, but they have received much less attention than the BDs. In this review, we discuss recent advances in the development of non-BD BET inhibitors, as well as their potential applications, advantages, and perspectives. The prospects of targeting phosphorylation hotspots and phosphorylation-dependent sites, ET, or IDR for governing BET-dependent transcription in cancer cells are outlined in Section 2, Section 3 and Section 4, and approaches to inhibiting non-BD-dependent BET interactions with viral proteins are summarized in Section 5. Currently available non-BD-targeting anticancer/antiviral BET inhibitors are summarized in Table 1.

## 2. Targeting the Phosphorylation-Dependent Interaction Domain and Its Potential Significance for Cancer Therapy

The affinity of BET family members for protein interactors is regulated through posttranslational modifications [35], primarily phosphorylation. BET proteins have multiple casein kinase II phosphorylation sites [18,36]. Most of them are clustered into the N-terminal cluster of phosphorylation sites (NPS) and the C-terminal one (CPS) (Figure 1A). To date, detailed studies of the dependence of BET activity on the NPS phosphorylation status have only been reported for BRD4. For other BET proteins, the effects of NPS phosphorylation are expected to be similar, considering the relatively high homology within the family, except for the C-terminal fragments.

**Figure 2:**
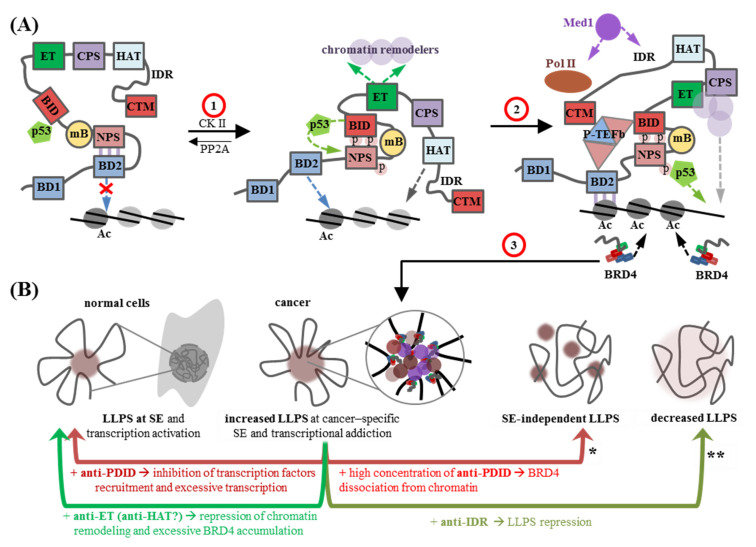
BRD4 as a target for cancer therapy. (**A**) Schematic representation of BRD4 contributions to normal transcription activation or transcriptional addiction in cancer. (1) NPS dephosphorylation by the phosphatase PP2A facilitates BRD4 autoinhibition via NPS-BD2 contacts that prevent BD2 from binding to acetylated histones. Casein kinase (CK) II-mediated phosphorylation of NPS releases BD2, thus affording BRD4 positioning at acetylated chromatin sites, while phosphorylated NPS forms contacts with BID, bringing BID partners, such as p53, to target chromatin sites. At the same time, BRD4 dimerizes in an mB-dependent manner (not indicated in the figure). This leads to local BRD4 accumulation. (2) The intrinsic HAT activity of BRD4 and its ET-dependent affinity for other epigenetic writers promotes additional acetylation of nearby histones and further chromatin remodeling, respectively. At lengthy hyperacetylated regions, such as SEs, the BRD4 concentration reaches the critical point sufficient for a phase transition (liquid–liquid phase separation, LLPS), namely, IDR-dependent co-separation with Med1. Transient interactions with Med1, either with LLPS (at SEs) or without LLPS (at common enhancers), bring BRD4-bound chromatin and associated transcription factors into proximity with Pol II, facilitating transcription initiation, while PDID-BID/CTM-dependent recruitment of P-TEFb enables Pol II phosphorylation and transcription elongation. (3) Aberrant BRD4 accumulation and at cancer-specific SEs underlies excessive LLPS and transcriptional activation. (**B**) Approaches to transcription regulation using BET-targeting/BET-derived therapeutics. The top panel represents differ types of BRD4-depedent LLPS, particularly in normal and cancer cells. The bottom panel summarizes approaches to preventing aberrant or excessive transcription using BET inhibitors. The colors of the arrows and legends correspond to the colors of the related (targeted) domains/motifs in Figure 1. * similar effects are observed for BBDi and SynGRs; ** similar effects are observed for BET-PROTACs.

In BRD4, dephosphorylated NPS (aa 484–506) binds BD2 (Figure 2A), rendering it inaccessible for interactions with acetylated histones H3/H4 [37] and some other acetylated proteins, such as the tumorigenic transcription factor TWIST1 [38] or triacetylated cyclin T1, the subunit of positive transcription elongation factor b (P-TEFb) [18]. BRD4 with dephosphorylated NPS resides in the nucleoplasm, whereas BRD4 with phosphorylated NPS is chromatin-bound [37]. Such a dependence of BRD4 localization and activity on the NPS phosphorylation status appears to prevent excessive or untimely transcription activation. The entire BD2-NPS region (aa 287–530) is commonly referred to as the phosphorylation-dependent interaction domain (PDID).

Phosphorylated NPS binds the BID (aa 524–579), promoting the exposure of the BID-preceding coiled-coil motif B (mB, aa 506–527), which assists in BRD4 dimerization [39]. NPS-bound BID attracts additional transcription factors, such as p53 [37] (Figure 2A). Normally, p53 regulates dozens of genes under responsive element-containing promoters in a context-dependent manner [40], eventually enabling cell cycle arrest in response to stress/damage. In cancer cells, BRD4 with NPS-bound BID recruits p53 to target specific genes, including the protooncogenes *c-Myc* and *c-Fos* [37].

The significant part of the PDID with phosphorylated NPS and BID (aa 351–598; this region is also termed BD2-B-BID), is also responsible for the intrinsic kinase activity of BRD4 [41]. This atypical kinase domain phosphorylates the Pol II C-terminal domain and several other ET domain binding substrates to eventually facilitate transcription elongation. Thus, PDID with phosphorylated NPS and active BID are essential for the multiple contributions of BRD4 to gene upregulation, particularly in cancer. Thus, phosphorylated NPS targeting ligands may turn out to be more potent and specific anticancer drugs than BD binders. In addition, hyperphosphorylation of BRD4 has been implicated in the long-term resistance of cancer cells (e.g., NUT midline carcinoma) to BBDi [42]. This resistance can theoretically be overcome by combining BBDi with PDID-targeting ligands.

Unlike canonical domains with well-defined folding, NPS and BID are IDRs [17], so NPS/BID-targeting ligands are typically developed by screening rather than structure-based design. For the first attempt to selectively target NPS, a combinatorial library of 14^4^ peptoids (oligo-N-substituted glycines) with artificial substituents was developed [32]. Screening of this “one bead one compound” library revealed two peptoids, **DC-1** and **DC-2** (Figure 1B), which were affine for phosphorylated NPS but not for other phosphoproteins, phosphorylated CPS, or PDID with dephosphorylated NPS. **DC-1** showed the highest level of selectivity in this respect but had some cross-reactivity with other components of the crude lysates of eukaryotic (insect) or bacterial cells.

Among the small-molecule inhibitors identified by high-throughput screening [17], compound **HTS-21** (Figure 1B) was the most promising ligand of phosphorylated NPS. Optimization of **HTS-21** by chemical modification led to the compound **SDU-071** (Figure 1B), for which the AlphaScreen assay revealed a particularly effective disruption of the BRD4 interaction with p53 (IC_50_ = 3.1 µM). **SDU-071** effectively suppresses cell proliferation, migration, and invasion by downregulating the expression of BRD4-targeted genes, such as *c-Myc*, and inducing cell cycle arrest and apoptosis. This was demonstrated in cultured MDA-MB-231 triple-negative breast cancer cells, and its antitumor activity was illustrated in vivo in an orthotopic mouse xenograft mammary tumor model [26] (Table 1).

To summarize, the currently available potent PDID inhibitors include phosphorylated NPS binding small-molecules.

## 3. Extra-Terminal Domain Targeting and Related Anticancer Drug Candidates

The highly conserved ET domain (>85% identity in BRD2/3/4 [27]) renders BET proteins affine to chromatin remodelers (Figure 2A), including the catalytic subunit (CHD4) of the nucleosome remodeling and deacetylase (NuRD) complex [43], the ATPase catalytic subunit of the INO80 chromatin remodeling complex [44], histone methyltransferases WHSC1 (NSD2) and WHSC1L1 (NSD3) [45], the truncated sex combs-like protein 1 (ASXL1) component of the Polycomb-repressive deubiquitinase complex [46], and the arginine demethylase and lysyl hydroxylase JMJD6 [47].

This ET-dependent ability to recruit epigenetic writers/erasers complements the BD-dependent BET functions as epigenetic readers during transcription elongation [48]. In particular, the recruitment of JMJD6 facilitates the activation of P-TEFb by BRD4 and the release of paused Pol II into productive elongation [49]. NSD3 recruitment promotes H3K36 methylation, thereby ensuring transcriptional fidelity [50,51]. In BRD2, BRD3, and BRDt, but not BRD4, the ET domain is flanked by the coiled-coil (CC) motif, and the ETCC module is thought to be responsible for BRD2/3-specific interactions, such as binding to RNA polymerase II-associated factor (PAF) [52]. Inhibition of ET/ETCC interactions with chromatin remodelers critical for oncogenesis (e.g., the breast cancer-associated JMJD6 [53]) may be a route to robust epigenetic drugs.

In contrast to the search for PDID inhibitors, which is mainly based on small-molecule library screening, the design of ET inhibitors originated from structural studies of the ET interaction landscape. ET consists of three α-helices forming an acidic ridge and a hydrophobic cleft [27]. NMR studies of the BRD4 ET domain (aa 600–678) in complex with peptides [28] revealed a consensus ET binding motif: KϕKϕ, where ϕ is a hydrophobic or an aromatic amino acid. This amphipathic motif binds ET by forming a two-stranded antiparallel β-sheet with the ET α2-α3 loop anchored to the hydrophobic cleft of the three-helix bundle. A similar study with the BRD3 ET domain (aa 571–644) gave the same results [27]. The authors referred to the BRD3 ET binding peptides as “KIKL-like sequences” and showed that their interaction with BRD3 was similar to that of KϕKϕ with BRD4. Further studies of ET with other peptide arrays confirmed these results for both BRD3 [54] and BRD4 [46]. Given that ET is conserved within the BET family, BRD2 and BRDt can be expected to bind KϕKϕ sequences in a similar manner [27]. These works formed the basis for the design of ET-targeting peptide ligands.

The first reported ET-targeting ligand was a 17-mer peptide derived from the ET-binding integrase of murine leukemia virus [29]. It showed high (K_D_ ~160 nM) binding affinity for BRD4 and outcompeted a number of chromatin remodelers, including NSD3. The relatively large size of the peptide limited its potential application. Molecular modeling was therefore used to identify a minimal set of essential contacts. In particular, the most likely binding sites were found using docking based on the Glide XP and Glide SP-Peptide protocols, the stability of the complexes was assessed by molecular dynamics, and the binding energy scoring was evaluated by General Born Surface Area (GBSA). The computational modeling yielded a truncated analogue LKIRL with similar binding efficiency. This pentapeptide effectively suppresses the proliferation of acute myeloid leukemia cells, as was demonstrated in the MOLM-13 cell line (Table 1). Another peptide inhibitor, **PiET** (RNQKFKCGE), was designed on the basis of JMJD6 and meant to act as a specific competitor of this tumorigenic protein [30]. **PiET** effectively disrupted the interaction between BRD4 and JMJD6 in vitro. It was then modified with a TAT transmembrane sequence, and the resulting cell-permeable peptide TAT-**PiET** prevented BRD4-JMJD6 interaction in cultured cells. Another modification was TAT-**PiET**-PROTAC, which degraded BRD4 in an ET domain-dependent manner. Both TAT-**PiET** and TAT-**PiET**-PROTAC were highly effective in suppressing breast cancer growth both in cultured cells (MCF7, T47D, MDA-MB-231, and BT549 cell lines) and in mouse tumor models (MCF7 and MDA-MB-231 cell-derived xenografts).

## 4. Targeting the C-Terminal IDR of BRD4 and Its Promise for Anticancer Drug Design

BET proteins have several intrinsically disordered regions (IDRs): NPS, BID, and CPS, which are present in all family members, and the long C-terminal IDR, which is characteristic of BRD4L [55]. IDRs are important drivers of liquid–liquid phase separation (LLPS) [56]. This process underlies the formation of intracellular biomolecular condensates (membraneless organelles) such as nucleoli, transcription/replication foci, reparation condensates or condensates at super-enhancers (SEs), and, to a lesser extent, at common enhancers and promoters [57,58,59]. BRD4 plays a prominent role at SEs, where it facilitates the accumulation and separation of the mediator complex and, together with Med1, scaffolds the condensates through its C-terminal IDR [10,60] (Figure 2A). The term “super-enhancer” was proposed in 2013 [59] and refers to a cluster of enhancers in close genomic proximity that cooperatively assemble a high density of the transcriptional apparatus [10]. In normal cells, SEs regulate the expression of genes that specify cell identity and functionally conform to cell type-specific biological processes. In cancer cells, key oncogenes are also regulated by SEs and BRD4-driven aberrant SE activation may trigger oncogenesis [61,62] (Figure 2B).

The BD-dependent affinity of BRD4 for acetylated histones contributes to LLPS indirectly and determines the location of the condensates: multi-acetylated chromatin fragments, such as SEs, act as BRD4 accumulation sites and LLPS nucleation sites. Displacing BRD4 from oncogene-controlling SEs with BBDi could alleviate transcriptional addiction in cancer [12]. This was demonstrated by the effect of pan-BBDi **JQ1** on multiple myeloma cells, where its addition caused a preferential loss of BRD4 at SEs, thus preventing BRD4-dependent recruitment of Med1 and P-TEFb and repressing SE-associated genes, including *c-Myc* [59]. The side effect was the appearance of SE-independent condensates [63], which could eventually lead to unpredictable expression changes.

One possible route to resolving condensates, rather than displacing them from acetylated chromatin, is the development of specific BRD4 C-terminal IDR-targeting ligands [64]. This approach has been undermined by recent evidence that overexpression of BRD4S, which lacks the C-terminal IDR, induces intracellular condensate formation more efficiently than the full-length isoform BRD4L [55]. A possible explanation for this apparent contradiction is the formation of different types of condensates with BRD4L and BRD4S. The former condensates are critically dependent on the C-terminal IDR of BRD4L and that of Med1 and are partially nucleosome-free due to the intrinsic histone acetyltransferase (HAT) activity of the BRD4 HAT domain (Figure 1A). In addition to the H3/H4 tails, it acetylates the K122 residue in the H3 globular part, weakening H3-DNA interactions and disrupting the octamer core, which eventually results in nucleosome eviction. In contrast, BRD4S lacks the HAT motif and cannot exclude nucleosomes [65,66], so the disordered N-terminal regions of histones may contribute to LLPS at BRD4S accumulation sites along with the relatively short BRD4S IDRs (NPS, BID, CPS). Overexpression of both BRD4L and BRD4S may be tumorigenic. Both isoforms promote drug resistance in ovarian cancer, according to recent data [67], although previous reports have emphasized the pathogenic role of BRD4S [68,69]. While both types of condensates are responsive to BBDi, those of BRD4L at cancer-specific SEs are also sensitive to anti-IDR LLPS disruptors (Figure 2B).

Non-selective LLPS disruptors, such as 1,6-hexanediol, which interferes with weak hydrophobic interactions and is commonly used to disassemble protein condensates in vitro and in cells, are highly toxic [70]. However, 1,6-hexanediol sensitivity studies are a convenient approach for LLPS verification and have been actively used to prove in vitro LLPS of BRD4S [55], of BRD4 C-terminal IDR and Med1 IDR [10], and in cellular LLPS at SEs [10]. Wang et. al. identified a natural product called **PCG** (Figure 1C) from *Polygonum cuspidatum* Sieb. et Zucc., a traditional Chinese medicinal herb that selectively binds BRD4, but not other BET proteins [31]. Using a series of truncated BRD4s, they found that **PCG** interacts with two segments of the BRD4 IDR, namely, P1 (aa 751–800) and P2 (aa 951–1031) (Figure 1A). The distinctive features of these segments are their proline-rich sequences, the general hydrophobicity and the lack of stable secondary structures. **PCG** interactions with the P1 and P2 segments induced BRD4 misfolding, disrupted phase separation in vitro, and converted dynamic nuclear condensates into insoluble aggregates, eventually repressing transcription of BRD4-dependent genes, including *c-Myc*. Its effect on gene expression in MDA-MB-231 and HeLa cells was comparable to that of a model BBDi JQ1 (Table 1).

## 5. Extra-Terminal Domain or Phosphorylation-Dependent Interaction Domain Targeting Ligands and Other Noncanonical BET Inhibitors as Antiviral Drug Candidates

**Figure 3:**
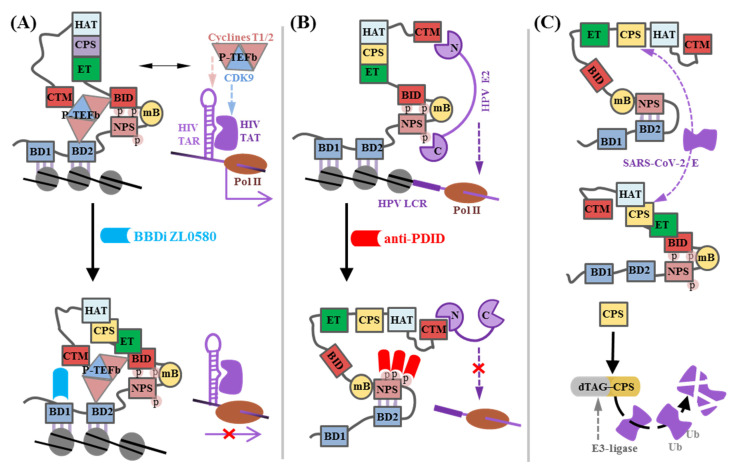
BRD4 as a target for antiviral therapy. (**A**) Schematic representation of BRD4 contributions to the HIV-1 life cycle. Top: HIV hijacks P-TEFb from phosphorylated BRD4 for transcriptional elongation. The P-TEFb components cyclin T1 and cyclin-dependent kinase 9 (CDK9) are recruited to the HIV-1 TAR hairpin and the TAR-binding protein Tat, respectively. Bottom: the BD1-specific noncanonical BBDi ZL0580 complements to the PDID-BID interactions with cyclin T-CDK9, thus stabilizing the entire complex and preventing P-TEFb redistribution to TAR-Tat. (**B**) Schematic representation of BRD4 contributions to the life cycle of high-risk oncogenic HPV16/18. Top: HPV16/18 E2 binds BRD4 at the long control region (LCR) of early HPV genes and repress their expression. The N-terminal domain of the HPV E2 protein binds CTM, promoting p-TEFb release from BRD4, and the C-terminal domain of E2 binds phosphorylated NPS. The latter interaction is crucial for E2-mediated repression of HPV promoter activity and ori-replication control. Bottom: anti-PDID ligands displace the E2 C-terminal domain from NPS-BID thereby disorganizing transcription and replication machinery of the viruses. (**C**) BET protein-based approach to inhibit SARS-CoV-2 viral assembly. Top: SARS-CoV-2 E protein binds BRD2 or BRD4 CPS domain independently of NPS phosphorylation status. Bottom: binding of the SARS-CoV-2 E protein with the CPS fused with the E3-ligase dTAG leads to degradation of the E protein.

Domains and motifs of the BET proteins that are critical for transcription activation or chromatin remodeling can be occupied by viral proteins in the infected host cell, because viruses exploit BET proteins to anchor their genome components to chromatin and compete with BET proteins for transcription factors (Figure 3). This makes BET proteins promising targets for antiviral therapy. One notable example of an antiviral BET inhibitor is **ZL0580** (Figure 1D), which interferes with the competition between host BRD4 and the TAT-bound transactivation response (TAR) RNA element of the HIV LTR for P-TEFb (Figure 3A). **ZL0580** can be termed a noncanonical specific BBDi because of its selectivity for BD1 of BRD4 (IC_50_ = 163 nM) over other BET proteins [71] and an unusual interaction mode. In silico analysis revealed that the binding site of **ZL0580** is distinct from the Kac-binding pocket of BD1 targeted by classical BBDi. Therefore, unlike classical BBDi, **ZL0580** does not disrupt BRD4 interactions with chromatin targets, but rather stabilizes the P-TEFb complex with PDID-BID, preventing P-TEFb redistribution to TAT-TAR. The antiviral activity of **ZL0580** was confirmed for both transcriptionally active and latent HIV in multiple cell models, including J-Lat cells, human PBMCs/primary CD4 T cells, and human myeloid cells/microglia [71].

The ET domains of BRD2 and BRD4 can be recognized by the Kaposi’s sarcoma-associated herpesvirus latent nuclear antigen (LANA). This enables the integration of the herpesvirus episome into host chromatin, leading to a decrease in BRD4-mediated gene activation and the cell cycle alteration [72]. ET domains of all human BET proteins are also recognized by the retroviral integrases of the gamma-Porcine Endogenous Retrovirus-A/C [73] and of the murine leukemia virus [54]. However, the binding mode differs from that of the consensus host ET-binding peptide (KϕKϕ). The murine leukemia virus integrase folds back onto itself to form a third antiparallel β-strand in complex with BRD4-ET. Interestingly, the affinity of this interaction is significantly higher than that reported for the consensus motif.

The C-terminal domain of the human papillomaviruses (HPV) sequence-specific transcription/replication factor E2 can bind BID, and the E2 N-terminal domain binds BRD4 CTM, while E2 of high-risk oncogenic HPV-16 and HPV-18 also binds phosphorylated NPS. The latter interaction is crucial for E2-mediated repression of HPV early genes, which are regulated by the long control region (LCR) with E2-binding sites, and ori-replication control [33] and can be disrupted by NPS inhibitors (Figure 3B). For instance, **DC-1** was shown to disrupt the interaction of NPS with E2 at low concentrations (20 µM) in tube- and in C33-A-based cellular models. Increasing **DC-1** concentrations also blocked the interaction of NPS with BID (IC_50_ = 27 µM) and the binding of BID to E2. However, it also enabled the interactions between the unleashed NPS and BD2. Therefore, at high (>330 µM) **DC-1** concentrations, BRD4 failed to recognize acetylated chromatin. Thus, PDID-targeting compounds offer a unique opportunity to block replication of cancer-associated high-risk HPV E2, but overdosing may prevent BRD4 from binding to its chromatin targets. Further high-throughput screening of a 200,000 compound library allowed for the authors to identify three small-molecule compounds (**14**, **90**, and **ZM145**, Figure 3A) that target phosphorylated NPS more selectively than **DC-1** [17]. These compounds exhibited a strong inhibition of high-risk HPV replication in a 3D organotypic raft culture of primary human keratinocytes by disrupting the interaction of NPS with HPV E2.

The CPSs of BRD2 and BRD4 (also known as Ser/Gln-rich sites or SEESs) have shown an affinity for the SARS-CoV-2 envelope (E) protein (Figure 3C), and the CPS-E interaction has been considered as a viral hijacking mechanism for transcriptional modification in host cells [34]. However, the underlying data were obtained using a cellular model with ectopic E expression. It remains to be clarified whether the E protein actually enters the nucleus during SARS-CoV-2 infection or whether the phosphorylation status of the BET protein affects binding. Despite significant homology with CPS, NPS contributes little to E recognition, probably because of its involvement in intramolecular contacts with BD2 or BID. BRD4 CPS was used to obtain the first SARS-CoV-2 E targeting PROTAC-dTAG-CPS, originally termed dTAG-SEED [34]. It reduced the level of ectopically expressed E protein in HEK293T cells (Table 1), but thorough verification of its antiviral activity remains to be performed.

## 6. Discussion

The examples discussed in this review suggest that non-BD-targeting ligands could be considered as anticancer agents for monotherapy or combination therapy. A potential advantage of monotherapy is the suppression of oncogene expression without global BRD4 redistribution by inhibiting the NPS-dependent recruitment of transcription factors, BD2-B-BID-dependent kinase activity, ET-dependent recruitment of chromatin remodelers, or IDR-dependent LLPS. Their combination with BBDi could prevent long-term drug resistance resulting from compensatory BRD4 activation through hyperphosphorylation. All of these approaches aim to restrict transcription at BRD4-dependent stages, from initiation to the onset of elongation. Productive elongation is dependent on the histone chaperones BRD2/3 and their binding to PAF, which can be inhibited by ETCC ligands.

The two key limitations to the development of non-BD targeting BET inhibitors are (i) the challenges of verifying their in vivo targets and the mechanisms of action and (ii) the difficulty of establishing effective screening systems [74]. The latter is particularly relevant for targeting NPS, BID, CPS, and the C-terminal part of BRD4, all of which are IDRs. The potential inhibitors of IDR-driven LLPS and the available inhibitor **PCG,** supposedly specific for Pro-rich motifs of the BRD4 C-terminal IDR, should be rigorously tested for interference with membraneless organelles other than condensates at SEs (e.g., speckles containing the Pro- and Gln-rich splicing factor SPQF [75]) to avoid global side effects. The cross-talk between domains and motifs (e.g., the PDID-dependent autoinhibition of BD2) is another source of potential side effects of non-BD ligands.

Further investigation of the BD-independent BET interactome may help to control side effects of known drug candidates and reveal new druggable sites. For example, a BD-independent interaction between BRD4 and GATA4 [76] has recently been reported to regulate the mitochondrial homeostasis in the adult heart, and the interaction interface is a potential target for pharmacological treatment of cardiovascular diseases. Another understudied potential target site in BET proteins is CPS. Its phosphorylation markedly reduces LLPS of BRD4 in vitro [55], but the significance of this effect in cells awaits further investigation.

One more unique site yet to be targeted in BRD4 is the histone acetyltransferase (HAT) catalytic motif (aa 1122–1161), which is specific for the full-length isoform. Apart from this motif in the C-terminal IDR, the HAT activity is mediated by the BD1-flanking acetyl-CoA binding site (aa 175–180) [64]. However, the primary mechanism of BRD4 HAT activity regulation is the switch to the C-terminal IDR-free BRD4S isoform by alternative splicing. This HAT activity is responsible for the ability of the BRD4L isoform to initiate the positive feedback loop of BRD4 accumulation by acetylating H3/H4 histone tails, which promotes chromatin decompaction and additional BET loading. Together with the BD2-B-BID-dependent intrinsic kinase activity towards Pol II CTD, CDK9, TAF7, and MYC [41], as well as the IDR-dependent ability to co-separate with Med1 at SEs [10], the intrinsic HAT activity underlies the pivotal role of BRD4 in transcription activation [66] and pro-tumorigenic effects [12]. Similar to PDID targeting, targeting the HAT motif may limit BRD4 accumulation at SEs rather than trigger its uncontrolled redistribution [63]. Guided redistribution of BRD4 can be achieved using synthetic genome readers/regulators (SynGRs), which comprise a sequence-specific DNA-binding motif and a BBDi-like ligand. This approach has been implemented to develop GAA repeat-targeting SynGRs [77,78]. Such SynGRs were effective in recruiting BET proteins, including BRD4, to frataxin gene and restoring frataxin transcription in cells derived from Friedreich’s ataxia patients.

Except for the BRD2/3/t-specific CC motif (the PAF binding site) and the C-terminal IDR of BRD4 (the LLPS driver), most of the non-BD targets are rather conserved within the BET family, so the respective ligands await thorough selectivity testing. Fine-tuning of these ligands may rely on computational approaches, similar to BBDi optimization [79,80,81]. For high complexity non-BD targets (ET and ETCC), the interaction maps could be obtained from molecular modeling or structural data, and residue-based free energy decomposition, in silico alanine screening, or analogous methods could be used to identify the essential contacts [82]. Low-complexity sequences (NPS, BID, CPS, and C-terminal IDR) are more challenging in terms of rational ligand design. However, new approaches to limit the conformational plasticity or multivalency of IDRs are under development [83] and will hopefully complement random screening.

In conclusion, non-BD targeting BET inhibitors represent a promising class of potential therapeutics. They show low toxicity and high anticancer/antiviral activity in cellular or mouse models (Table 1). However, most of them require additional testing and fine-tuning to ensure selectivity.

## Figures and Tables

**Table 1:** Noncanonical BET inhibitors and BET-derived drug candidates.

Inhibitor Code	Target (BET Domain)	Design Strategy	Binding Assays	Activity, Model	Refs.
**Anticancer**					
HTS-21	Phosphorylated NPS	High-throughput screening.	AlphaScreen assay (inhibition of BRD4 PDID-p53 interactions, IC_50_ ≈ 50 μM).	Suppression of proliferation (IC_50_ = 38 ± 1 μM, 72 h; IC_50_ = 36 ± 7 μM, 96 h), migration, and invasion of triple-negative breast cancer cells (line MDA-MB-231) via induction of cell cycle arrest and apoptosis. Suppression of tumor growth (37% at 50 mg/kg, 21 days) in the MDA-MB-231 orthotopic mouse xenograft mammary tumor model; no substantial systemic toxicity.	[17,26]
SDU-071	Phosphorylated NPS	Rational design (HTS-21 optimization).	Computational docking studies, AlphaScreen assay (inhibition of BRD4 PDID-p53 interactions, IC_50_ ≈ 3 μM).	Suppression of proliferation (IC_50_ = 10.5 ± 0.3 μM, 72 h; IC_50_ = 12.6 ± 0.4 μM, 96 h), migration, and invasion of triple-negative breast cancer cells (line MDA-MB-231) via induction of cell cycle arrest and apoptosis. Suppression tumor growth (49% at 50 mg/kg, 21 days) in the MDA-MB-231 orthotopic mouse xenograft mammary tumor model, no substantial systematic toxicity.	[26]
KϕKϕ/KIKL-like peptides	ET	Rational design (NMR-based analysis of ET complexes with its native interactors).	Isothermal titration calorimetry (interaction of NSD3 fragment with BRD4-ET, K_D_ = 141 µM).	Antiproliferative activity against acute myeloid leukemia cells expressing short NSD3 isoform is expected.	[27,28]
LKIRL	ET	Rational design (optimization of KIKL-like peptides using molecular dynamic simulations).	Surface plasmon resonance (interaction with BRD4-ET, K_D_ = 145 nM).	Suppression of proliferation (≈50-fold) of acute myeloid leukemia cells (line MOLM-13).	[29]
(TAT)-PiET-(PROTAC)	ET	Rational design (NMR-based analysis).	Surface plasmon resonance (interaction with BRD4-ET, K_D_ = 90 nM).	Suppression of proliferation (MCF7, T47D, MDA-MB-231, and BT549 cell lines, EC50 = 8 ± 1, 13 ± 1, 5 ± 1, and 32 ± 1 μM, respectively) and invasion of breast cancer cells; minimal effects on HEK293T cells. Suppression of tumor growth (>50% at 25 mg/kg every 2 days) in the MCF7 and MDA-MB-231 orthotopic mouse xenograft mammary tumor model, no substantial systematic toxicity.	[30]
PCG	P1 and P2	Revealed accidentally.	SDS-PAGE followed by western blotting.	LLPS assays; suppression of BRD4-dependent gene expression in triple-negative breast cancer cells (line MDA-MB-231). Suppression of proliferation of HeLa and MDA-MB-231 cells at high concentration (>100 µM).	[31]
**Antiviral**					
DC-1/2	Phosphorylated NPS	Screening of a peptoid library.	Fluorescence polarization assays (interaction of fluo-DC-1 with PDID, K_D_ ≈ 50–100 µM).	Suppression of HPV16/18 replication is expected based on the inhibition of BRD4-E2 interactions in C-33A cells.	[32,33]
14, 90, and ZM145	Phosphorylated NPS	High-throughput screening.	AlphaScreen assay (disruption of BRD4 PDID-16E2 interactions, IC_50_ = 5.1 μM for 14, IC_50_ = 1.6 μM for 90, and IC_50_ = 2.5 μM for ZM145).	Suppression of HPV18 replication in a 3D organotypic raft culture of primary human keratinocytes (10 μM of 14 and 3 μM of 90 or ZM145 reduced HPV18 DNA levels by ≥90%) with no morphologically discernible adverse effects (ZM145 exhibited the least cytotoxicity towards proliferating W12E cells).	[17]
KϕKϕ/KIKL-like peptides	ET	Rational design (NMR-based analysis of ET complexes with its native interactors).	NMR titration (BRD4-ET with KSHV LANA peptide (aa 1133–1144), K_D_ = 635 µM).	Suppression of Kaposi’s sarcoma-associated herpesvirus replication is expected.	[27,28]
dTAG-CPS	BET-derived PROTAC	Rational design.	Co-immunoprecipitation, proximity ligation assay.	Decrease in SARS-CoV-2 E protein levels (≈50%) in HEK293T cells.	[34]

## Data Availability

No new data were created or analyzed in this study. Data sharing is not applicable to this article.
